# Sciatic neuropathy following endovascular treatment of a limb vascular malformation

**DOI:** 10.1186/1749-7221-1-8

**Published:** 2006-12-15

**Authors:** John P Ney, William Shih, Mark E Landau

**Affiliations:** 1 Madigan Army Medical Center, Neurology Service, 9040A Fitzsimmons Dr. Tacoma, WA 98431, USA; 2 Naval Medical Center, Neurology Service, 34800 Bob Wilson Drive San Diego, CA 92134, USA; 3 Walter Reed Army Medical Center Department of Neurology, 6900 Georgia Ave., NW, Bldg #2, Washington, DC 20307, USA

## Abstract

**Background:**

Endovascular therapy for vascular malformations is one of the treatment options for limb vascular malformations.

**Case presentation:**

A patient with a vascular malformation of the hip developed ipsilateral leg weakness immediately after endovascular embolization and sclerotherapy. Clinical and electrodiagnostic findings later confirmed an incomplete sciatic neuropathy.

**Conclusion:**

We propose that endovascular treatment compromised the patient's sciatic nerve either through direct neurotoxicity of the sclerosing agent or ischemic injury.

## Background

Vascular malformations of the limbs can present with a variety of symptoms. Diagnostic investigations uncover a range of anatomical complexity. Tools in the management of these lesions include direct surgical intervention, percutaneous sclerotherapy, and catheter-guided angiographic approaches. [1] Endovascular sclerotherapy is a more selective method aimed at reducing blood flow within the vascular malformation. Identification of feeding vessels to muscles and nerves are essential in reducing the risk of vascular compromise to these structures. We report a patient with a large arteriovenous malformation (AVM) of the hip undergoing selective endovascular embolosclerotherapy who developed a post-procedure sciatic neuropathy.

## Case presentation

**Figure 1:**
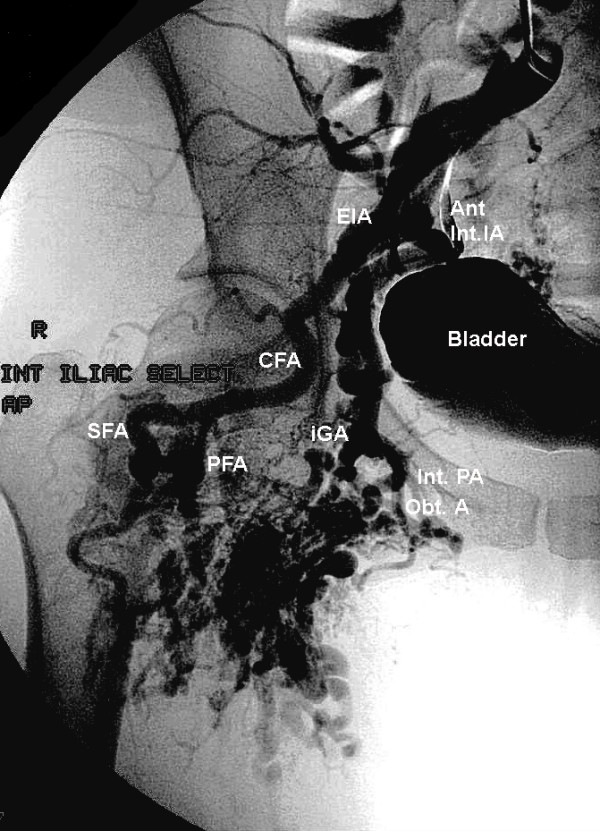
Conventional arteriogram-Anteroposterior views of right hip and pelvis vasculature. Iliac vessels prior to embolosclerotherapy and coil placement. The anterior division of the internal iliac is engorged, feeding into the AVM. The profunda femoris artery contributes to the AVM as well.

**Figure 2:**
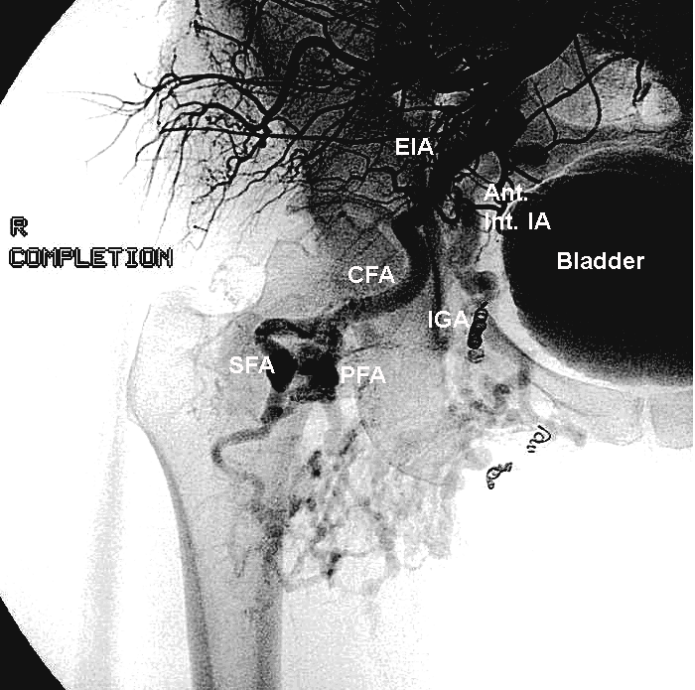
Conventional arteriogram-Anteroposterior views of right hip and pelvis vasculature. Diminution of flow to the AVM post-procedure. Note reduction in size of anterior internal iliac branches including inferior gluteal artery. (Abbreviations-EIA-external iliac artery, Ant. Int. IA-anterior division of internal iliac artery, CFA-common femoral artery, SFA-superficial femoral artery, PFA-profunda femoris artery, IGA-inferior gluteal artery, Int. PA-internal pudendal artery, Obt. A-obturator artery.)

A 47-year old with a large right lower extremity AVM, identified during evaluation for exertional hip and thigh pain, underwent her fourth endovascular procedure. A 5 French sheath was placed in the left common femoral artery using standard Seldinger technique. A pigtail catheter was inserted to the level of L-2 and the right common iliac artery was selected using a guide wire. After initial arteriography (Figure 1), major feeding vessels for the medial branches of the vascular malformation deriving from the anterior division of the internal iliac artery were identified and embolized using tornado coils. Three milliliters of 100% ethanol was directed into the vascular malformation. Completion arteriogram showed total reduction in size and flow of the AVM by 75%. (Figure 2)

Immediately following the procedure, the patient had new complaints of right leg weakness. Physical examination revealed complete inability to dorsiflex or invert the right foot. She had 4/5 MRC scale strength of plantar flexion, foot inversion, and knee flexion, with preservation of strength in hip girdle muscles and knee extensors. Her deep tendon reflexes were 2/4 throughout with the exception of a 1/4 right ankle jerk. She was anesthetic on her right lateral leg. Subsequent MRI of her lumbar spine and CT of her abdomen and pelvis failed to disclose any acute pathological process. Six weeks later, a nerve conduction study showed absent right superficial peroneal and sural sensory responses, and absent peroneal (recording from the extensor digitorum brevis) and tibial (recording from the abductor hallucis) motor responses. Needle electromyography (EMG) demonstrated muscle membrane instability, manifested by fibrillation potential and positive sharp waves in the right tibialis anterior, medial gastrocnemius, and semitendinosus. No motor unit potentials (MUPs) were observed in the right tibialis anterior. Small, polyphasic MUPs with decreased recruitment were noted in the semitendinosus and gastocnemius. Needle EMG of the gluteus medius and lumbar paraspinal region was normal.

At the time of electrodiagnosis, she had significant atrophy in her right calf, anterior leg and hamstring muscles. Based on the clinical and electrodiagnostic examinations, she was diagnosed with a severe, but incomplete lesion of the right sciatic nerve. Magnetic resonance neurography was unavailable at our institution. Despite vigorous physical therapy, she had not recovered strength since the initial post-endovascular procedure examination. She had not undergone any subsequent nerve repair procedures at the time of this writing.

## Conclusion

Embolosclerotherapy is recommended in isolation or as part of a multi-staged approach for treatment of appendicular vascular malformations. Where the vascular malformation is deep with extensive muscle or bony involvement, embolosclerotherapy is preferred to surgical treatments which may leave the patient with greater disability. [2] Ethanol or N-butyl cyanoacrylate are the sclerosing agents most often utilized. Absolute ethanol is toxic to vascular endothelium, inducing thrombosis in affected vessels. [3] Delivery of the agent to the nidus of the lesion is essential in reducing systemic side effects, such as pulmonary hypertension. [1] Catheter-guided techniques are recommended for arteriovenous malformations, whereas percutaneous sclerotherapy is sufficient for venous malformations. [3] Success as defined by reduction in the size of the vascular malformation and diminished associated symptoms has been reported in up to 94.7% of sessions in percutaneous and catheter-guided techniques followed for an average of 10.6 months. [1]

Ethanol sclerotherapy may damage peripheral nerves either through direct nerve toxicity or ischemia. [4] Lee, et al [5] reported a series of eighty-seven patients who underwent a total of 399 sessions of percutaneous ethanol sclerotherapy. Five nerve palsies were reported; three facial and two peroneal. A mechanism of injury was not postulated.

Reports of catheter-guided endovascular treatments complicated by nerve injury are scant. Liang, et al reported recurrent laryngeal nerve injury resulting in vocal cord paralysis after embolization of patent ductus arteriosus in three infants. [6] Quinn, et al, published a small series of patients who underwent catheter-guided cisplatin chemotherapy for pelvic cancers that developed femoral and sciatic nerve lesions [7]. Stent-graft of an abdominal aortic aneurysm with embolization of iliac arteries was complicated by lower extremity weakness and fecal incontinence in one patient [8].

We contend that our patient's nerve injury was the result of ischemia or toxicity to the sciatic nerve. The acuity and severity of the lesion are consistent with either of these pathophysiologies. The sciatic nerve is supplied by vessels ultimately derived from the internal iliac artery. The inferior gluteal artery arises from the anterior division of the internal iliac, descending in the pelvis to give off the sciatic artery. This small vessel maintains the sciatic nerve blood supply through a network of *vaso nervorum*. Collateral vessels from the iliolumbar, lateral sacral, obturator, and internal pudendal arteries contribute to the vascular supply of the region as well. [7] Ischemia resulting in nerve injury would involve the inferior gluteal artery primarily, with secondary compromise of other anastamotic vessels.

Our patient had distal coil placement and a neurotoxic vascular sclerosing agent infused into her right anterior internal iliac artery, causing ischemia to the affected vascular territory, including the sciatic nerve. Her three prior infusions and coil placements may have prevented collateral blood flow. Although limb vasculature malformations can cause local muscle necrosis, [9] we found nerve conduction and electromyographic abnormalities in a sciatic nerve distribution far distal to her lesion. The acute onset of her weakness renders a pre-existing sciatic neuropathy unlikely.

Embolosclerotherapy for limb vascular malformations remains a valuable treatment for a difficult condition. Awareness of the relationship of the vascular malformation and its blood supply to nervous structures may prevent similar neurologic complications of this procedure in the future.

## Abbreviations

MRI, magnetic resonance imaging; CT, computed tomography, AVM, arteriovenous malformation; MRC, medical research council;

## Financial support

None

## Financial disclosures

None

## Competing interests

The author(s) declare that they have no competing interests.

## Authors' contributions

JN performed the literature search, wrote the manuscript, prepared the images, and submitted the paper. WS acquired the clinical data, edited the manuscript, and edited the paper. ML identified the case, acquired the data, and edited the paper.

## Disclaimer

The opinions or assertions contained herein are the private views of the authors and are not to be construed as official or as reflecting the views of the Department of the Army or the Department of Defense.

## References

[B1] Lee BB Bergan JJ Advanced management of congenital vascular malformations: a multidisciplinary approach Cardiovasc Surg 2002 1 523 33 10.1016/S0967-2109(02)00072-8 12453680

[B2] Yakes WF Haas DK Parker SH Gibson MD Hopper KD Mulligan JS Pevsner PH Johns JC Jr Carter TE Symptomatic vascular malformations: ethanol embolotherapy Radiology 1989 1 1059 66 2916057 10.1148/radiology.170.3.2916057

[B3] Yakes WF Pevsner P Reed M Donohue HJ Ghaed N Serial embolizations of an extremity arteriovenous malformation with alcohol via direct percutaneous puncture AJR Am J Roentgenol 1986 1 1038 40 3485898 10.2214/ajr.146.5.1038

[B4] Weber J Belov ST, Loose DA, Weber J Embolizing materials and catheter techniques for angiotherapeutic management of AVM Vascular Malformations 1989 Reinbek: Einhorn-Presse Verlag GmbH 252 60

[B5] Lee BB Do YS Byun HS Choo IW Kim DI Huh SH Advanced management of venous malformation with ethanol sclerotherapy: mid-term results J Vasc Surg 2003 1 533 8 10.1067/mva.2003.91 12618688

[B6] Liang CD Ko SF Huang SC Huang CF Niu CK Vocal cord paralysis after transcatheter coil embolization of patent ductus arteriosus Am Heart J 2003 1 367 71 10.1016/S0002-8703(03)00125-X 12891209

[B7] Quinn SF Frau DM Saff GN Kavanagh J Roberts W Cavanagh D Clark RA Neurologic complications of pelvic intraarterial chemoembolization performed with collagen material and cisplatin Radiology 1988 1 55 7 3347746 10.1148/radiology.167.1.3347746

[B8] Kwok PC Chung TK Chong LC Chan SC Wong WK Chan MK Chu WS Neurologic injury after endovascular stent-graft and bilateral internal iliac artery embolization for infrarenal abdominal aortic aneurysm J Vasc Interv Radiol 2001 1 761 3 11389230 10.1016/s1051-0443(07)61450-x

[B9] Breugem CC Maas M van der Horst CM Magnetic resonance imaging findings of vascular malformations of the lower extremity Plast Reconstr Surg 2001 1 878 84 10.1097/00006534-200109150-00010 11547142

